# Automated annotation of developmental stages of *Drosophila* embryos in images containing spatial patterns of expression

**DOI:** 10.1093/bioinformatics/btt648

**Published:** 2013-12-03

**Authors:** Lei Yuan, Cheng Pan, Shuiwang Ji, Michael McCutchan, Zhi-Hua Zhou, Stuart J. Newfeld, Sudhir Kumar, Jieping Ye

**Affiliations:** ^1^School of Computing, Informatics, and Decision Systems Engineering, ^2^Center for Evolutionary Medicine and Informatics, The Biodesign Institute, Arizona State University, Tempe, AZ 85287, USA, ^3^National Key Laboratory for Novel Software Technology, Nanjing University, Nanjing 210023, China, ^4^School of Life Sciences, Arizona State University, Tempe, AZ 85287, USA and ^5^Center of Excellence in Genomic Medicine Research, King Abdulaziz University, Jeddah, Saudi Arabia

## Abstract

**Motivation:** *Drosophila melanogaster* is a major model organism for investigating the function and interconnection of animal genes in the earliest stages of embryogenesis. Today, images capturing *Drosophila* gene expression patterns are being produced at a higher throughput than ever before. The analysis of spatial patterns of gene expression is most biologically meaningful when images from a similar time point during development are compared. Thus, the critical first step is to determine the developmental stage of an embryo. This information is also needed to observe and analyze expression changes over developmental time. Currently, developmental stages (time) of embryos in images capturing spatial expression pattern are annotated manually, which is time- and labor-intensive. Embryos are often designated into stage ranges, making the information on developmental time course. This makes downstream analyses inefficient and biological interpretations of similarities and differences in spatial expression patterns challenging, particularly when using automated tools for analyzing expression patterns of large number of images.

**Results**: Here, we present a new computational approach to annotate developmental stage for *Drosophila* embryos in the gene expression images. In an analysis of 3724 images, the new approach shows high accuracy in predicting the developmental stage correctly (79%). In addition, it provides a stage score that enables one to more finely annotate each embryo so that they are divided into early and late periods of development within standard stage demarcations. Stage scores for all images containing expression patterns of the same gene enable a direct way to view expression changes over developmental time for any gene. We show that the genomewide-expression-maps generated using images from embryos in refined stages illuminate global gene activities and changes much better, and more refined stage annotations improve our ability to better interpret results when expression pattern matches are discovered between genes.

**Availability and implementation:** The software package is available for download at: http://www.public.asu.edu/∼jye02/Software/Fly-Project/.

**Contact:** jieping.ye@asu.edu

**Supplementary information:** Supplementary data are available at *Bioinformatics* online.

## 1 INTRODUCTION

Increasingly higher throughput bio-imaging technologies are enabling scientists to capture the spatiotemporal patterns of gene expression, which promises to generate a more comprehensive picture of genome function and interaction (Cardona and Tomancak, 2012; Peng *et al.*, 2007; Walter *et al.*, 2010; Yakoby *et al.*, 2008). Today, gene expression and protein localization patterns are being captured with unprecedented spatial resolution in numerous model organisms. For example, more than one hundred thousand images of gene expression patterns from early embryogenesis are available for *Drosophila melanogaster* (fruit fly) (Lécuyer *et al.*, 2007; Tomancak *et al.*, 2002). These images are a treasure trove for identifying co-expressed and co-regulated genes and for tracing the changes in a gene’s expression over time (Lécuyer *et al.*, 2007; Tomancak *et al.*, 2002). Knowledge gained from analyses of these *Drosophila* expression patterns is widely important because a large number of genes involved in fruit fly development are commonly found in animal kingdom (Levine and Davidson, 2005; Simpson, 2002; Weiss, 2005). Consequently, many of the inferences made from studies of fruit flies have been shown to apply to humans and other species (Chen and Crowther, 2012; Kumar, 2001; Levine and Davidson, 2005; Miller *et al.*, 2013; Simpson, 2002; Weiss, 2005; Williams *et al.*, 2012). Overall, research efforts into the spatial and temporal characteristics of gene expression patterns of *Drosophila* have been at the leading edge of scientific investigations into the fundamental principles of animal development (Kalinka *et al.*, 2010; Konikoff *et al.*, 2012; Osterfield *et al.*, 2013; Tomancak *et al.*, 2002; Walter *et al.*, 2010).

**Fig. 1.**
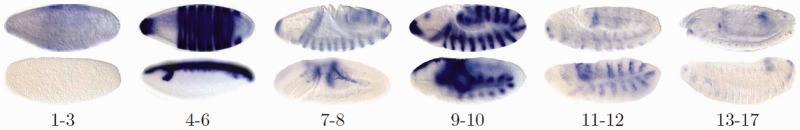
Sample images from the BDGP. It has the largest collection of images for early as well as late stages. The images in BDGP are grouped into six stage ranges: 1–3, 4–6, 7–8, 9–10, 11–12 and 13–17 (Tomancak *et al.*, 2002, 2007)

The comparative analysis of gene expression patterns is most biologically meaningful when images from a similar time point are compared (Campos-Ortega and Hartenstein, 1997). Based on morphological landmarks, the continuous process of *Drosophila* embryogenesis is traditionally divided into a series of Stages (1, 2, 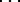, 17) (Kumar *et al.*, 2002). However, the standard practice of manually inspecting images containing spatial patterns is a rate-limiting step, especially when it has to be done for large number of images produced by high-throughput techniques. Images generated in some high-throughput experiments are currently given stage range assignments (e.g. 4–6) rather than individual stages (Fig. 1). As the original developmental stage delineations are based on major morphological events in the fruit fly development, it is, in principle, possible to distinguish embryos in images at the level of individual stages (Ji *et al.,* 2008; Ye *et al.,* 2006, 2008). However, previous methods (Cai *et al.*, 2012; Meng and Shyu, 2011) only predict stage ranges, and no methods currently exist to provide specific stage annotations for *Drosophila* embryos. Furthermore, no approach currently exists to annotate developmental stage for an embryo on a continuous numerical basis, which would be more biologically realistic because development is a continuous process that is reflected in the output of the high-throughput experiments. Visually, it is possible to scan a set of embryonic expressions and arrange them into a progression of gene expression, which informs us about the change and direction of spatial expression over time. This indicates need for a system that has the ability to assign more finely graded stage information that enables one to conduct biological discovery using images with higher resolution of stage similarity. In this article, we report one such computational system and show how it enhances visualization and scientific discovery.

## 2 MATERIALS AND METHODS

**Fig. 2.**
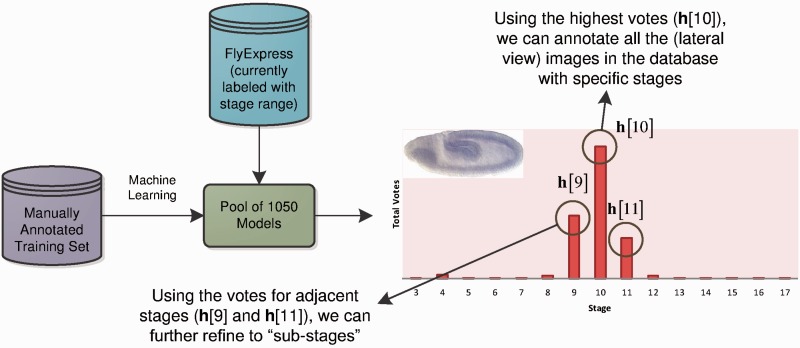
Overview of our stage annotation system. By learning from a training dataset with manually labeled stage information, we build a pool of 1050 classification models. We then apply this pool to the unlabeled images in our *FlyExpress* database, providing a histogram of voting values for each image. The histogram is then used to annotate the image with a specific stage, as well as a more refined ‘sub-stage’ and numerical-based ‘stage score’

To develop an automated annotation system, we began by building a comprehensive training set, in which development experts identified images that were exemplar for each developmental stage defined in (Campos-Ortega and Hartenstein, 1997). This constituted our initial training/testing set and contained 3724 images (all in lateral view) such that there were >200 images for each stage considered (Table 1). We applied machine learning (Bishop *et al.*, 2006) to develop a pool of 1050 classification models to discriminate among stages. For any image, all 1050 models are applied to generate a stage prediction, which produces the voting histogram (Fig. 2). This histogram is used to generate estimates of embryo stage annotation at various levels of granularity. In the simplest case, we classify an embryo to be of stage S if a majority of models designated the image to be in Stage S. For example, Stage 10 gets the highest number of votes, and thus it is assigned to the embryo in the image under consideration (Fig. 2). This histogram also shows that the number of votes for Stage 9 is higher than that for Stage 11, which enables a finer stage designation (early Stage 10, 10E) for this embryo. We also generate a stage score (SS) using the frequencies in the voting histogram to incorporate non-symmetry of the distribution and relative size of the most frequent peaks. For the example in Figure 2, 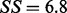. These stage scores can be used to order images based on embryonic developmental time or produce finer grade stage annotations.

**Table 1.** Number of annotated BDGP images for each developmental stage

Stage	No. of images	Stage	No. of images
1–3	250	11	246
4	251	12	255
5	274	13	251
6	224	14	252
7	236	15	232
8	260	16	243
9	248	17	254
10	248	Total	3724

This collection of images is manually annotated with precise stage labels. The orientation of all images in this study is standardized, and the size is scaled to 128 by 320 pixels.

The rest of this section is organized as follows. In Section 2.1, we discuss the training set we built as ‘ground truth’ for our system. We then present the various machine learning methods used to create a big pool of models in Section 2.2. Finally, we introduce the annotation of previously unseen images in Section 2.3.

### 2.1 Training set acquisition

To develop an automated annotation system, a key component is to build a comprehensive training set, in which each entity (in our case, images of gene expression in *Drosophila* embryo) is associated with the ‘accurate’ annotation (in our case, the corresponding stage). By learning from the training set, a system will extract critical information from the images that discriminates the developmental stages from each other and uses the extracted knowledge to build classifiers for predicting the stage of previously unseen images. We have manually annotated a collection of images with precise stage labels for 3724 standardized Berkeley Drosophila Genome Project (BDGP) images (in lateral view) in *FlyExpress*. The detailed numbers of labeled images are listed in Table 1. Embryogenesis in *Drosophila* starts with 13 rapid nuclear divisions after fertilization. Thus, the only morphological difference across the first stage range (Stages 1–3) is the number of nuclei, a feature not visible with the microscopy used by the BDGP consortium. Therefore, they are considered as a single stage (Stage 3) in this work. The alignment and orientation of all images in this study are standardized using a semi-automated pipeline, and the size is scaled to 128 by 320 pixels (Konikoff *et al.*, 2012).

### 2.2 Model pool construction

The key of a successful voting system is to build a pool of diverse classification models, each with reasonably good performance. In this section, we will first introduce the feature extraction process and then present different ways of building classification models by using the underlying structure of the features.

#### 2.2.1 Feature construction

To make images from different stages easier for computational models to distinguish, appropriate feature extraction is critical. Log Gabor filters (Daugman, 1980; Field, 1987) have been shown to offer the best simultaneous localization of spatial and frequency information with an arbitrary bandwidth. They are particularly suitable for our study, as the features distinguishing between different stages should focus on the general morphology of the embryo as well as subtle textures. In the frequency domain, the log Gabor function with respect to radius (r) and angle (θ) can be described by:

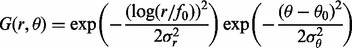

where *f*_0_ is the filter’s center frequency, 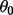 is the filter’s orientation and 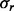 and 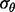 are the corresponding standard deviations. By choosing different values of *f*_0_ and 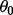, one can construct filters with different wavelet scales and orientations.

**Fig. 3.**
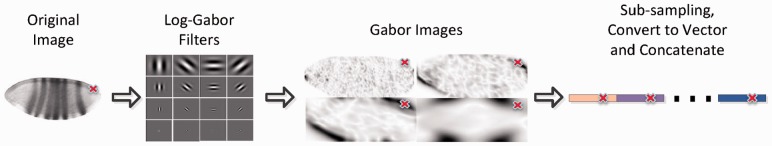
Illustration of the feature extraction process. The standardized image is first processed by a series of log-Gabor filters, resulting in 24 Gabor images. These Gabor images are then down-sampled and concatenated into a single feature vector, which is the final representation of the original image. As indicated by the cross in the figure, one sub-block of the original image corresponds to 24 features in the feature vector, one for each Gabor image

The procedure of our feature construction is illustrated in Figure 3. First, we converted the color image to gray scale. We then used log Gabor filters with four different wavelet scales and six different filter orientations to extract the texture information. Hence, 24 Gabor images were obtained from the filtering operation. Next, we divided each of the Gabor images into 640 sub-blocks of size 8 by 8, and the mean values were used to represent each of the sub-blocks. The 24 sub-sampled Gabor images were then converted to vectors, which were concatenated together as the feature vector for the original image. Thus, the dimension of the final feature vector is 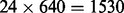.

#### 2.2.2 Preliminary on linear classifiers

#### 

The feature construction step maps the images into a feature space, with each dimension corresponding to a specific Gabor feature. We can then denote the training set as 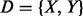, where 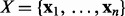 is the feature vector of the annotated images, 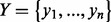 is the corresponding stage and *n* is the number of training samples. In our study, we apply linear classifiers on this high-dimensional classification problem, and apply the one-versus-the-rest (Bishop *et al.*, 2006) method to convert the multiclass classification problem into a series of binary class problems. Therefore, only binary linear classifiers will be discussed in the rest of this section. Specifically, a binary linear classifier takes the linear combination of the feature vector x of a sample to make the prediction:
(1)
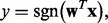

where 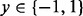 is the decision or the predicted ‘label’ of 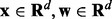 is the weight vector of the classifier that needs to be learned from the training data, and 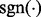 is the sign function.

Learning a linear classifier is to pursuit the optimal weight vector **w** on the training set, which can be formulated as the following optimization problem:
(2)
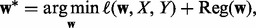

where 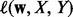 is the loss function measuring the discrepancy between the prediction and the ground truth for the training samples, and 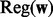 is a regularization term designed to improve the generalization performance of the classifier. The regularization term can be used to impose specific structures on the weight vector; and it will be discussed in detail in the following subsection. Three common loss functions are used in this study:

- Least square loss (Bishop *et al.*, 2006; Hastie *et al.*, 2009) (e.g. ridge regression):

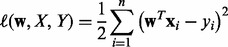

- Logistic loss (Bishop *et al.*, 2006; Hastie *et al.*, 2009) (e.g. logistic regression):

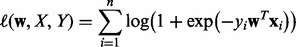

- Hinge loss (Bishop *et al.*, 2006; Vapnik, 2000) (e.g. support vector machine or SVM):

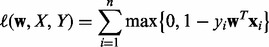



#### 2.2.3 Exploiting the underlying sparse structure

For high-dimensional small sample size problems such as the one in our study, 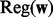 in Equation (2) plays a critical role in alleviating over-fitting and improving generalization performance. A common choice (e.g. in ridge regression and SVM) of the regularization term is: 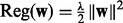.

An alternative way of addressing the high-dimensional problem is feature selection. In the rest of this subsection, we will discuss 3 variants of sparsity-inducing regularizations (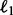 norm, 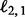 norm and 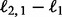 norm) that can impose different types of sparsity patterns on the solution of Equation (2) and lead to simultaneous classification and feature selection (Ye and Liu, 2012).

From Equation (1), one characteristic of a linear classifier is that if we set a certain entry of **w** to be 0, it is equivalent to removing the corresponding feature. This motivates us to introduce the 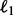 regularization (Tibshirani, 1996):

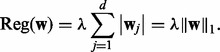



The 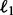 regularization (also called Lasso) performs feature selection and classification in a unified formulation. It has been applied successfully in various applications (Liu *et al.*, 2009). However, Lasso does not make full use of the underlying structure of our data. Specifically, as shown in our feature extraction process illustrated in Figure 3, each region of the image is associated with 24 features, one for each of the 24 different log-Gabor filters. Thus, the features can be naturally partitioned into distinct groups, one for each region of the image. It is then natural to apply group Lasso (Yuan and Lin, 2006), which can be applied to select feature groups, i.e. image regions. Assume that we partition the index of the features into *S* disjoint groups 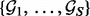, one for each region, such that 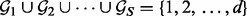. We can then obtain the 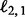 norm (also called group Lasso) regularization as follows:

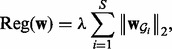

where 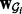 is the weight vector restricted to the i-th group of features, and λ is the parameter that controls the group sparsity.

When we use the 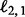 norm regularization to perform feature selection, all features from the same group will be selected simultaneously. Thus, only the ‘between-group sparsity’ is considered. However, some features from a selected group may be irrelevant to our prediction. In this case, the 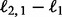 norm regularization (called sparse group Lasso) (Friedman *et al.*, 2010; Liu and Ye, 2010) can be applied, which simultaneously achieves the ‘between-group’ sparsity based on the 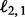 norm and the ‘within-group’ sparsity based on the 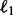 norm as follows:

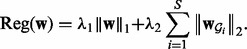



#### 2.2.4 Constructing a pool of diverse classifiers

The key idea of a successful voting system is to have a large and diverse pool of models, each of them with reasonable prediction power. In this study, we applied SVM with linear kernels from the LIBLINEAR (Fan *et al.*, 2008) package and six sparse learning algorithms (Lasso, group Lasso and sparse group Lasso with least square and logistic loss) from the SLEP (Liu *et al.*, 2009) package. We then partition the annotated dataset into two disjoint sets, namely, the ‘training set’ where linear classifiers are learned and the ‘validation set’ where the performance of the learned classifiers can be evaluated. Five different training set ratios (from 50 to 90%) are used to partition the dataset and for each ratio, 30 random partitions are generated. Each combination of classification algorithm and training set partition results in a distinct classification model.

In terms of classification algorithms, we find that all seven algorithms perform comparably with the three sparse learning methods using logistic loss achieving slightly better performance. The best cross-validation accuracy is *79.82* ± *1.67%*, which is achieved by sparse logistic regression with logistic loss and 90% of data as training. For our 15-class (Stages 3–17) classification problem, an accuracy of 80% is reasonably good. We also find that the validation accuracy generally increases as more samples are used in training, but the increase is not that significant after 70% of the annotated data (about 2600 images) are used for training. This indicates that the annotated dataset has an adequate size.

In addition to obtaining a collection of ‘reasonable’ models, we also need the models to be diverse such that the majority voting of the pool will provide robust results for unseen subjects. We calculate the average rate that at least one of the algorithms does not agree with the others, and find that the disagreement rate varies from 30 to 20% as the training ratio increases (refer to Supplementary Materials for detailed results on individual classifier performance as well as disagreement rate). Therefore, we have built a pool of 1050 (7 algorithms times, 5 training ratios times, 30 random partitions) diverse models, each of which achieves reasonably good classification performance.

### 2.3 Voting for stage annotation and beyond

In this subsection, we will discuss in detail the voting scheme we designed for annotating the remaining BDGP images in our *FlyExpress* database.

#### 2.3.1 Stage annotation by majority voting

For a given unlabeled image, we denote the prediction vector for this image based on the i-th model as 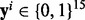, where 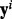 is a 15D binary vector indicating the stage prediction of the i-th model. Specifically, 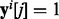 indicates that the i-th model determines that this image belongs to the j-th stage. We also assign a ‘confidence level’ of the current model as a^i^, which is set to be the classification accuracy of this model on the validation set. We then summarize all the predictions from the 1050 models and obtain a prediction histogram defined as 
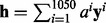
. Then, the entry with the highest voting will be the stage assigned by the ensemble of the pool of models. That is, the final annotation is defined as 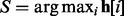.

#### 2.3.2 Sub-stage annotation and decimal-based embryo ordering

To illustrate our method of refining stage annotation to sub-stages and the decimal-based embryo ordering scheme, we first provide an example of the prediction histogram for a specific image in Figure 2. In our current system, only images assigned to Stages 4–16 have refined stage annotation.

As expected, Stage 10 gets the most votes among all 15 stages, and therefore this image will be annotated as Stage 10. We then compare the voting scores for the two adjacent Stages 9 and 11 and observe that 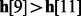. Therefore, according to our system, this Stage 10 image is more similar to Stage 9 compared with Stage 11. Thus, we will annotate this image as Stage 10E (early 10).

In addition to the order information of the prediction histogram, we can assign a continuous stage value for the image. Using Figure 2 as an example, we calculate the ‘stage score’ for this image as:

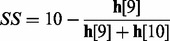

The intuition is as follows: the higher value of 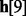 with respect to 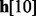 is, the ‘earlier’ this embryo is among all the Stage 10E images. This decimal stage value can only be used to suggest a relative order within each sub-stage. For example, in terms of developmental time, a Stage 7.9 image is not necessarily closer to Stage 8 than a Stage 6.7 image is to Stage 7.

With the help of the embryo ordering scheme, we can obtain even more refined stages. For example, we can further divide Stage 10E into three sub-sub-stages as follows: first, we sort all the decimal stage values of all the images assigned to Stage 10E. We then evenly split the sorted images into three groups, with the first group annotated as Stage 10E-a, second as 10E-b and third as 10E-c.

## 3 RESULTS AND DISCUSSION

We estimated the cross-validation performance of the annotation system in correctly assigning a specific stage (*S*) for the 3724 annotated images first. This produced an accuracy of 79%, with the highest accuracy observed for Stage 7 (89%) and the lowest accuracy for Stage 10 (44%). This may attribute to the fact that the differences between Stages 9 and 10 are small as they correspond to the slow phase of germ band movement.

For evaluating the performance of our method on independent data at a large scale, we generated the annotation *S* for 36 802 images (lateral views) obtained from the *FlyExpress* database (Kumar *et al.*, 2011). A stage assignment was deemed to be correct if *S* was within the stage range provided by the source BDGP (Tomancak *et al.*, 2002, 2007). That is, if an image was annotated as Stage 7 by our system (*S* = 7) and BDGP annotated it as stage range 7–8, then the annotation was considered to be correct. In this case, the accuracy of our annotations was 86.6%, with the highest accuracy seen for stage range 4–6 (96.9%) and the lowest for stage range 9–10 (74.9%). Visual inspection of mistakes revealed that a handful of images were not lateral views. Cai *et al.* (2012) reported an 85.2% cross-validation accuracy using 5414 images, whereas Ye *et al.* (2008) achieved 87.8% for just the 3 early stage ranges. Compared with previous results, our system is accurate in terms of predicting the stage ranges for all 36 802 images.

We also performed another independent evaluation by randomly selecting 140 images from Stages 4–17. We asked a domain expert to manually annotate these randomly selected images with specific stages (S) and more refined stages [e.g. Early Stage 10 (10E), late Stage 7 (7L)]. Of these, manual annotations were not provided by experts for 23 images because they were too out-of-focus to annotate or not lateral (mislabeled in the database). For the remaining 117 images, computational and manual annotations matched *81%* of the times, which is similar to the accuracy observed for the training set. At the level of sub-stages, manual and computational annotations matched *73.5%* of the time. Overall, we found that the computational prediction is within one sub-stage of the expert developmental biologists’ annotation for *93%* of the images tested. Therefore, the computational predictions can provide an excellent set of initial annotations.

### 3.1 Improving similar expression pattern retrieval

**Fig. 4.**
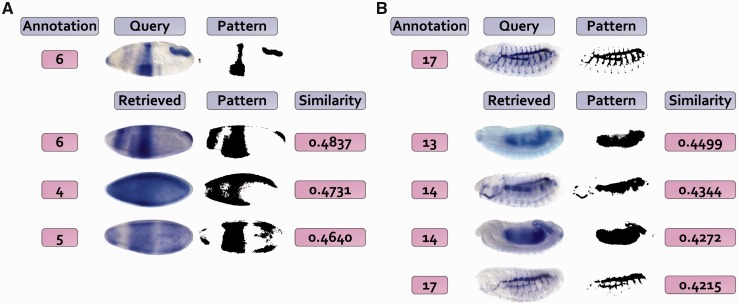
Examples of refining image retrieval results using stage annotation. Two example query images are used, with the left one (**A**) from the Kr gene and right one (**B**) from Gasp. The top matches from the *FlyExpress* lateral BDGP images are listed, with corresponding pattern as well as similarity values. The annotated stage from our system is presented on the left of each expression image

Within the *FlyExpress* database, we provide a tool for identifying similar gene expression patterns for a given query image (Kumar *et al.*, 2002). As the images in *FlyExpress* are assigned to a stage range, the search can only be done within a particular stage range. However, the comparison of gene expression is most biologically meaningful when the embryos are from similar developmental time points, which means that the use of specific stage would be useful to improving the interpretation of matches. We present two example cases where the use of specific and refined stages leads to better biological insights (Fig. 4). In Figure 4A, an expression profile of *srp* gene from stage range 4–6 is used to query for the best matching patterns. It produces results from many different genes within the same stage range. A view of the specific stage enables one to quickly realize that the query image was from Stage 6 and that many of the resulting patterns are from earlier stages (e.g. 4 and 5). So, by incorporating specific stage information, the user would have received results from Stage 6 only, which would have been more relevant. A similar situation exists for the second case (Fig. 4B), where the expression of Gasp from stage range 13–17 is used to query the database. Results in this case show spurious overlaps with many much earlier stage images (e.g. 13, 14), which have been included simply because of rather coarse stage annotations available. Therefore, we plan to provide users with an option in *FlyExpress* to view results that potentially represent the best matches that come from the closest predicted stage.

### 3.2 Genomewide-expression-maps with refined stage information

**Fig. 5.**
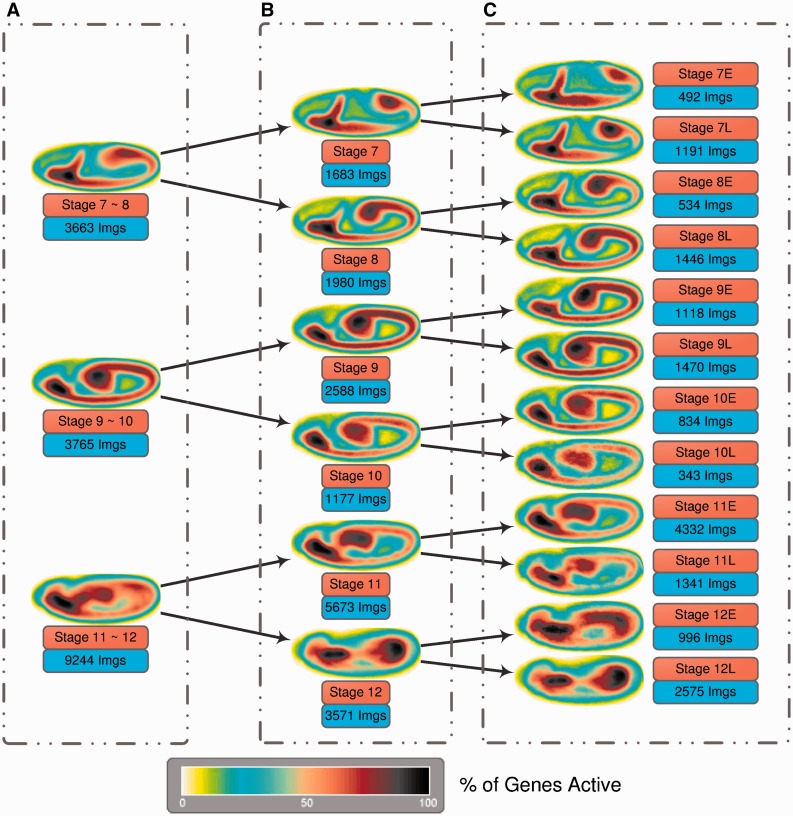
Stages 7–12 GEMs generated by using only the stage range information [(**A**), left column], the predicted stage information [(**B**), middle column] and the sub-stage information [(**C**), right column]. The total number of images involved for creating each individual GEM is also reported

**Fig. 6.**
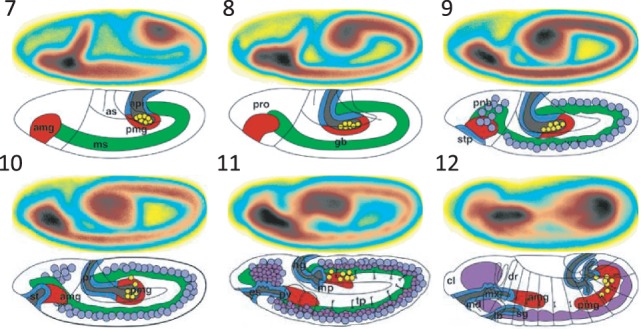
GEMs obtained from automatically annotated lateral BDGP images (Stages 7–12) compared with previously published overview images of stage development

Using the predicted stage information for 36 802 images (lateral views) obtained from the *FlyExpress* database (Kumar *et al.*, 2011), we created genomewide-expression-maps (GEMs) that are generated by aggregating and normalizing all spatial gene expression patterns from the same stage (Konikoff *et al.*, 2012; Kumar *et al.*, 2011). In Figure 5, we demonstrate how the use of increasingly refined stage information makes the global views of gene activities increasingly more informative. The results are arranged from top to bottom for images classified by BDGP in Stages 7–8, 9–10 and 11–12 (see Supplementary Materials for other stage ranges). In Figure 5A, GEMS for stage range 7–8 lack the information that the germ band is initially more posterior in position and moves toward the anterior, which is easily revealed when images from stage range 7–8 are separated into Stages 7 and 8. This trend is further illuminated when the stages are further refined into early and late parts (Fig. 5C). Comparing the Hartenstein (1993) images side by side with these GEMs confirms this trend (Fig. 6). Increasingly more refined trend is seen for Stages 9–10 and 11–12 as shown in Figure 5 (top to bottom in the right column), such that one quickly gets a sense of the developmental progression illuminated by gene expression patterns. These results indicate that the automated stage annotations work well and that refined stages will enable scientists to identify better sets of co-expressed genes.

We also predicted stage score for each image and then build GEMs at an even higher resolution than those in Figure 5, which shows how global gene activities vary over developmental time. In our supplemental materials, we provide a short video made by dividing each stage into 8 sub-sub-stages (‘BDGP_GEMs.avi’). In addition to categorizing embryo images into finer sub-stages, our stage score can help to sort all embryo images based on their estimated developmental time (refer to Supplementary Materials for more results on embryo sorting). This will add great functionalities to our current *FlyExpress* database, and a preliminary version is already included in our iPhone app (Kumar *et al.*, 2012).

### 3.3 More on model ensemble

In our final annotation system, all 1050 models are used to form the ensemble. One interesting question to ask is: is it truly beneficial to include all of them? In this subsection, we use the aforementioned independent evaluation dataset to validate our choice of large number of models.

First, we show that combining different classification algorithms is essential for the success of model ensemble. We predict the stages of the images from the evaluation set using the ensemble of different subset of methods, and the results are summarized in Table 2. Apart from the ensemble of all methods, we test three other scenarios: SVM models alone, sparse models alone and SVM models plus sparse models with logistic loss. Formally, we define the criteria as follows:

- **Sub-stage Accuracy** (Acc_0.5_). Only the images that are annotated with the correct sub-stage are considered accurate. For example, if an ‘early stage 7’ image is annotated as Stage 7E by our system, then the annotation is considered correct
- **Stage Accuracy** (Acc_Stage)_. The images that are annotated with the correct stage are considered accurate. For example, if an ‘early stage 7’ image is annotated as Stage 7E or Stage 7L by our system, then the annotation is considered correct
- **Plus-Minus-Sub-stage Accuracy** (Acc_±0.5_). The images that are annotated with a sub-stage which is at most ‘a sub-stage away’ from the manually annotated sub-stage are considered accurate. For example, if an ‘early stage 7’ image is annotated as Stage 6L, Stage 7E or Stage 7L by our system, then the annotation is considered correct.

As we can see from Table 2, neither SVM models nor sparse models yield competitive results, while the best performance is achieved by combining all of them together. This is especially true for the side-stage accuracy, where a large number of diverse models are essential for accurately predicting if an image is from the early or late part of a certain stage. Additional discussions such as the effects of ensemble pruning (Zhou, 2012) can be found in the Supplementary Materials.

**Table 2.** Performance evaluation of model ensemble using different subsets of the learning algorithms when stage range information is not available

Methods	Acc_0.5_ (%)	Acc_Stage_ (%)	Acc_±0.5_ (%)
SVM + sparse algorithms	73.50	81.20	93.16
SVM	52.99	76.07	91.45
Sparse algorithms	68.38	82.05	94.02
SVM + logistic algorithms	65.81	80.34	94.02

Three evaluation criteria are used, namely, the sub-stage accuracy (Acc_0.5_), the stage accuracy (Acc_Stage)_ and the plus-minus-half accuracy (Acc_±0.5_).

## CONCLUSION

In this article, we propose an automated system for the developmental stage annotation of *Drosophila* embryo gene expression images. A pool of 1050 classification models is constructed using a variety of state-of-the-art sparse learning algorithms. Based on this model pool, we design a voting scheme which not only produces accurate stage annotation but also a stage score for each embryo. This stage score can be used to more finely annotate each embryo into early and late periods of developmental stage. We use this system to annotate 36 802 images (lateral view) from the *FlyExpress* database, and show that the refined stage and sub-stage annotations greatly improve our ability to view global gene activities and to interpret matching expression patterns. Our current system is designed for size and orientation standardized images in the *FlyExpress* database. To extend our system for annotating non-standardized images (e.g. disoriented ones) will be an interesting future direction.

## Supplementary Material

Supplementary Data
