# Supplementary material for: Automated annotation of developmental stages of Drosophila embryos in images containing spatial patterns of expression
Source: Bioinformatics. 2013 Dec 3;30(2):266–73. doi: 10.1093/bioinformatics/btt648 (PMC3892688; doi:10.1093/bioinformatics/btt648)
Supplement: Supplementary Data [file supp_30_2_266__index.html]

Automated Annotation of Developmental Stages of Drosophila Embryos in Images Containing Spatial Patterns of Expression — Automated annotation of developmental stages of Drosophila embryos in images containing spatial patterns of expression — Automated annotation of developmental stages of Drosophila embryos in images containing spatial patterns of expression — Supplementary Data 

# Automated annotation of developmental stages of *Drosophila* embryos in images containing spatial patterns of expression

## Supplementary Data

files

**Files in this Data Supplement:**

- Supplementary Data - pdf file
- Supplementary Data - avi file
- Supplementary Data - avi file
