# Supplementary material for: Automated annotation of developmental stages of Drosophila embryos in images containing spatial patterns of expression
Source: Bioinformatics. 2013 Dec 3;30(2):266–73. doi: 10.1093/bioinformatics/btt648 (PMC3892688; doi:10.1093/bioinformatics/btt648)
Supplement: Supplementary Data [file supp_btt648_Suppliment-Stage.pdf]

Supplement to "Automated Annotation of Developmental Stages of Drosophila Embryos in Images Containing Spatial Patterns of Expression"

1 Embryogenesis as a Continuous Process

With the proposed majority voting scheme, we can annotate all the lateral BDGP images in our FlyExpress database with precise stages. This is a significant refinement from the currently available stage-range information. However, embryogenesis is a continuous process and a natural question to ask is: based on current information (training set annotated with stages), can we provide further refinement? Here we will present some intermediate results that provide us with suggestions to move beyond stage annotation to even further refinement. We first summarize the voting confidence level for all the models on the training data set. For example, for an image that is known to be from stage 4, we will calculate the actual predictions (they may vary from stage 3 to stage 17) from all the models. We then repeat the process for all the labeled images for stage 4, so that we will generate a "voting distribution" for images known to be stage 4. The voting distribution for all 15 stages is illustrated in Figure 1.

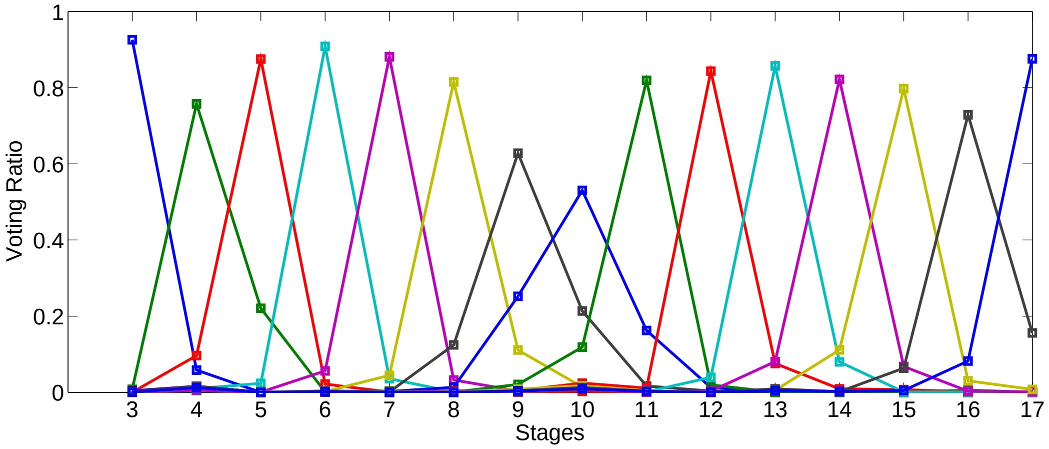

Figure 1: Summary of total voting for each stage in the labeled data set. For each stage in the training dataset, we summarize the predicted stages from all the models on all the images to obtain a voting distribution for that stage. The voting distribution will always peak at the corresponding stage.

We can see from Figure 1 that the highest ratio always appears in the corresponding stage, which is intuitive since all the models yield reasonable performance. More interestingly, the second and third highest ratios always appear in the adjacent stages. For example, in the distribution for stage 9 (black line with a peak at stage 9), the second and third highest voting ratio are stages 10 and 8 respectively. This shows that when a model disagrees with the actual stage annotation, it will mostly assign that image to an adjacent stage. This corresponds perfectly to the continuity of

the embryogenesis process, and motivates us to make additional use of the prediction histogram **h** to further refine the stage annotation.

2 Stage Score Based Embryo Sorting

In the FlyExpress database, one can search for all of the expression pattern images for a specific gene. Currently, the results are sorted by the annotated stage range. With the help of our embryo ordering scheme, one can sort all of the expression patterns according to the developmental time point suggested by our system. This enables us to examine how the expression pattern for a certain gene changes over time. We use three genes (twi, tkv and gt) as examples, and their sorted images are summarized in Figure 2, Figure 3 and Figure 4, respectively. Note that our embryo ordering scheme can only be used to determine the relative order within the same sub-stage. For example, a stage 7.9 image is not necessarily closer to stage 8 than a stage 6.7 image is to stage 7 (refer to the "Material and Method" section for details). In addition, we also generate video clips for a given gene by using each image as a frame. We include a video made with tkv images in our supplemental materials ("tkv.avi").

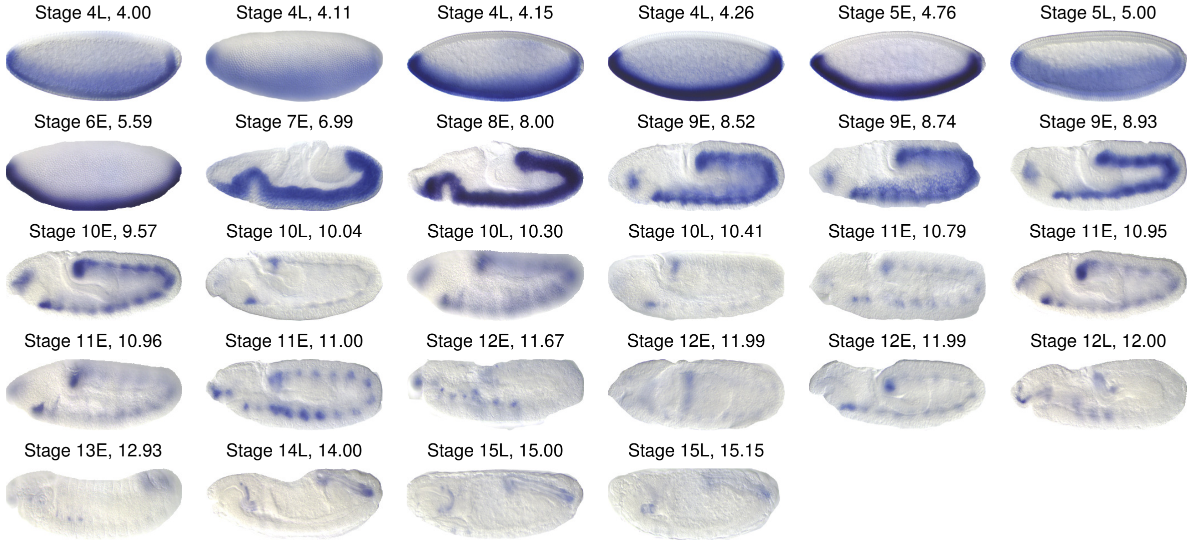

Figure 2: Lateral BDGP images from gene twi, sorted by decimal-stage annotations. The annotated stages as well as decimal-stage scores are reported on the top of each expression image.

3 Genomewide-Expression-Maps

For completeness, we include here the Genomewide-Expression-Maps generated for stages and sub-stages 4-6 and 13-16 in Figure 5 and Figure 6.

4 Performance of Individual Classifiers

In our model pool, we applied SVM with linear kernels from the LIBLINEAR (Fan et al., 2008) package, and 6 sparse learning algorithms (Lasso, group Lasso and sparse group Lasso with least

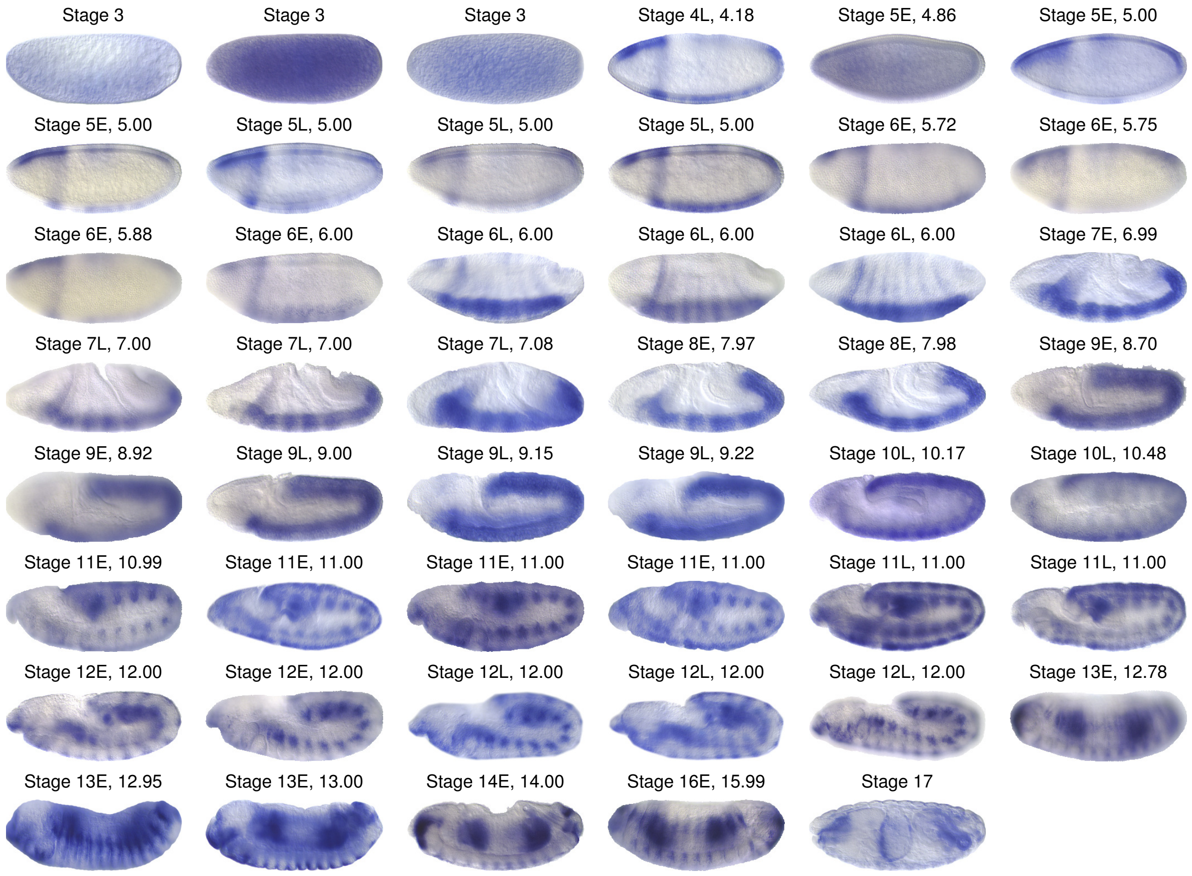

Figure 3: Lateral BDGP images from gene tkv, sorted by decimal-stage annotations. The annotated stages as well as decimal-stage scores are reported on the top of each expression image.

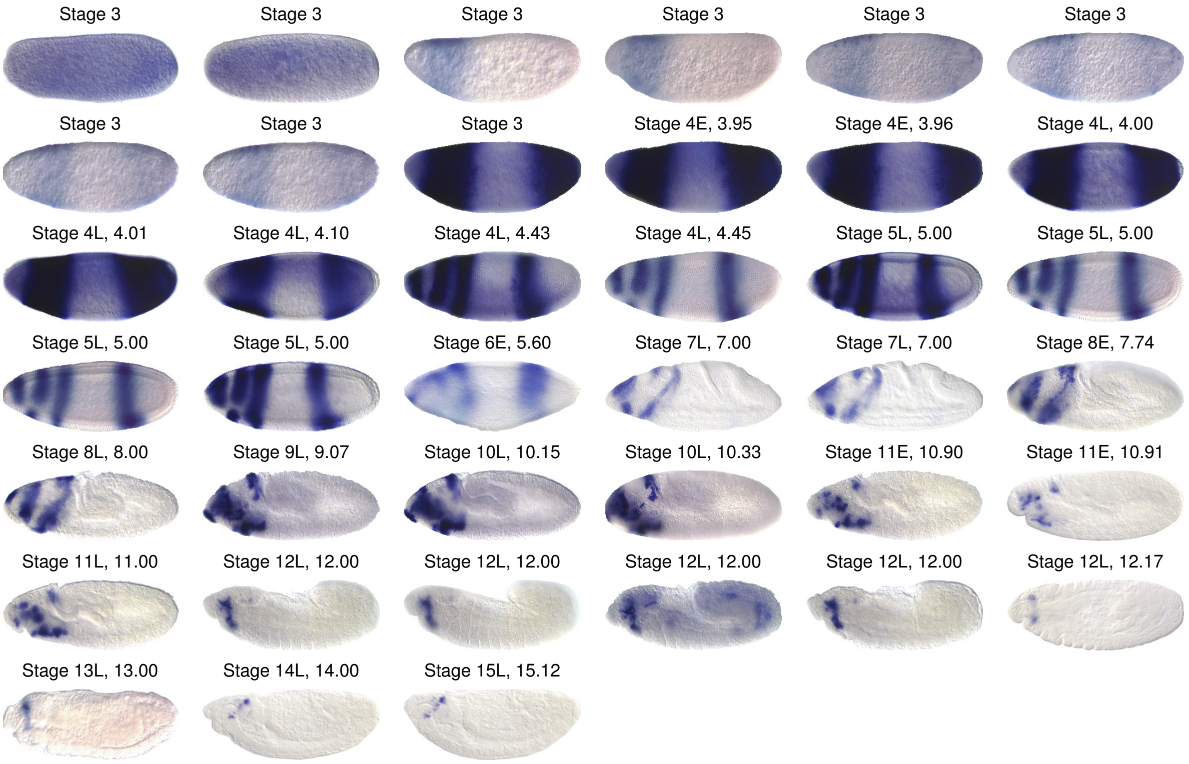

Figure 4: Lateral BDGP images from gene gt, sorted by decimal-stage annotations. The annotated stages as well as decimal-stage scores are reported on the top of each expression image.

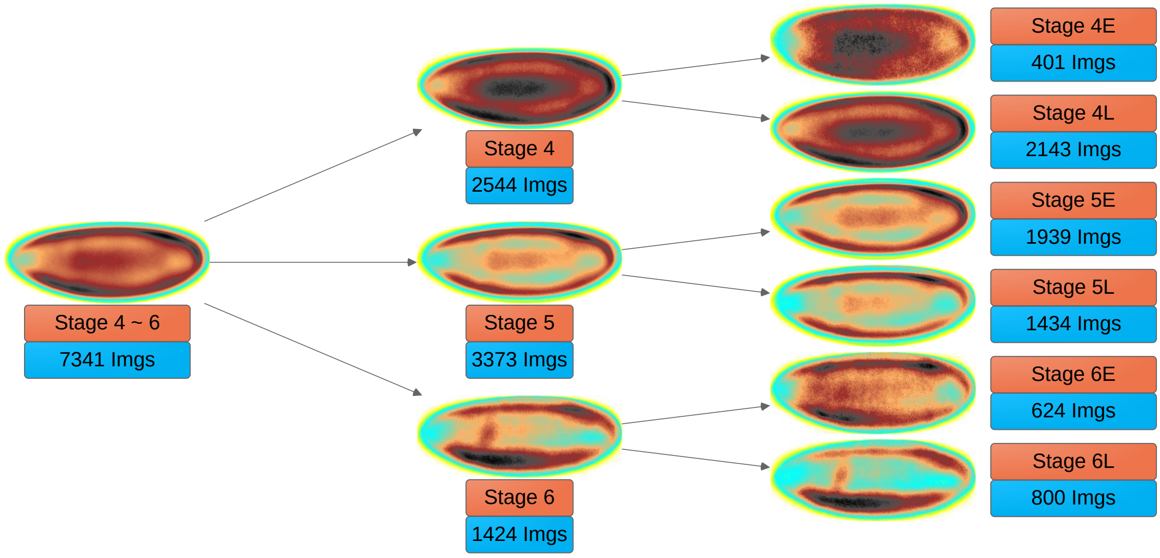

Figure 5: Stage 4 - 6 GEMs generated by using only the stage range information (left column), the predicted stage information (middle column) and the sub-stage information (right column). The total number of images involved for creating each individual GEM is also reported.

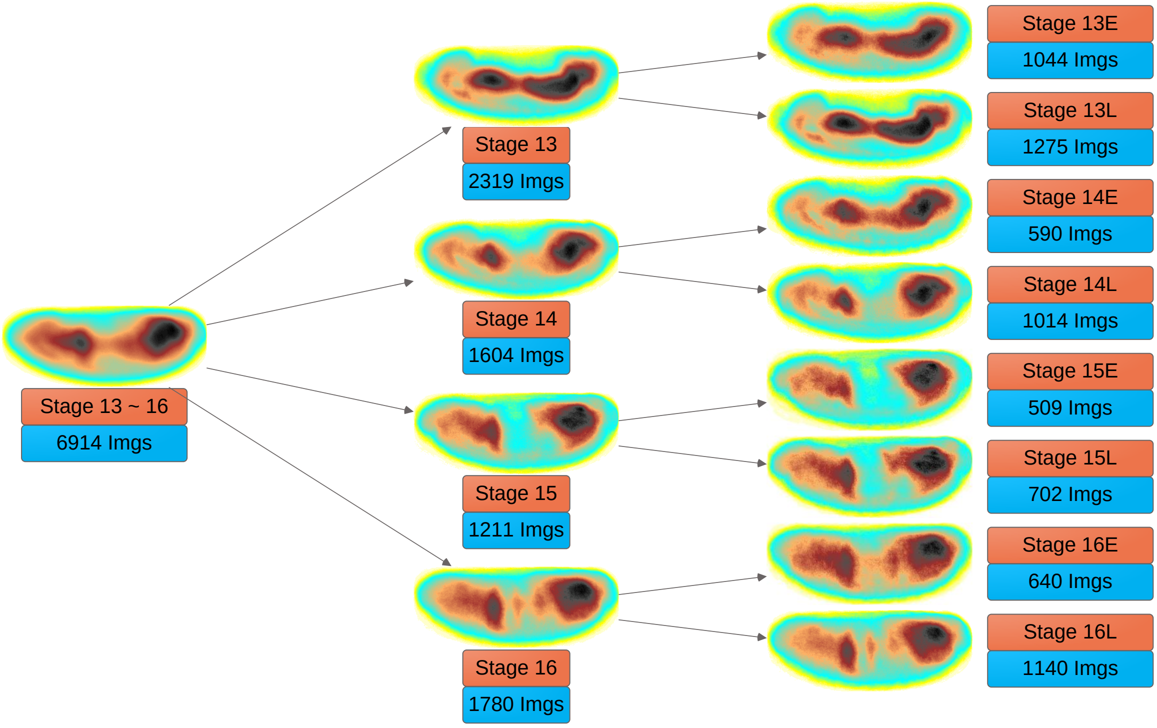

Figure 6: Stage 13 - 16 GEMs generated by using only the stage range information (left column), the predicted stage information (middle column) and the sub-stage information (right column). The total number of images involved for creating each individual GEM is also reported.

square and logistic loss) from the SLEP (Liu et al., 2009) package. We then partition the annotated data set into two disjoint sets, namely, the "training set" where linear classifiers are learned and the "validation set" where the performance of the learned classifiers can be evaluated. Five different training set ratios (from 50% to 90%) are used to partition the data set and for each ratio, 30 random partitions are generated. The average performance in terms of accuracy on the validation sets for different classification algorithms and training ratios is shown in Table 1.

Table 1: Average and standard deviation of classification accuracy (%) on the validation set for different algorithms and different training ratios, when stage range information is not available. 7 algorithms are evaluated, including SVM with linear kernel (SVM) and 6 sparse learning methods: Lasso (lasso), group Lasso (glasso), sparse group Lasso (sglasso) with least square loss (least) and logistic loss (log).

Ratio	50%	60%	70%	80%	90%
SVM	79.12 ± 0.81	79.10 ± 0.74	78.89 ± 1.01	78.79 ± 1.32	77.07 ± 2.02
lasso(least)	73.64 ± 1.80	74.89 ± 1.23	75.85 ± 1.35	77.33 ± 1.70	78.19 ± 2.04
glasso(least)	74.72 ± 1.31	75.08 ± 1.53	76.22 ± 1.41	77.77 ± 1.62	77.95 ± 1.77
sglasso(least)	75.29 ± 1.63	76.33 ± 1.16	77.02 ± 1.16	77.95 ± 1.71	77.70 ± 1.81
lasso(log)	78.34 ± 0.77	78.93 ± 0.81	79.27 ± 1.14	79.55 ± 1.72	79.79 ± 1.88
glasso(log)	78.61 ± 0.94	78.93 ± 0.91	79.40 ± 1.17	79.68 ± 1.49	79.87 ± 1.68
sglasso(log)	78.36 ± 0.98	78.81 ± 0.92	79.18 ± 1.05	79.49 ± 1.32	79.82 ± 1.67

As we can see from Table 1, all 7 classifiers perform comparably and the three sparse learning methods using logistic loss perform slightly better. For our 15-class (stages 3 to 17) classification problem, an accuracy of 80% is reasonably good. We can also see that the validation accuracy generally increases as more samples are used in training, but the increase is not that significant after 70% of the annotated data (about 2600 images) are used for training. This indicates that the annotated data set has an adequate size. We can also notice an increase in standard deviation with more training samples, which is intuitive since the size of the validation set is reduced.

In addition to obtaining a collection of "reasonable" models, we also need the models to be diverse such that the majority voting of the pool will provide robust results for unseen subjects. For each different training ratio, we calculated the average rate that at least one of the algorithms does not agree with the others, and the results are summarized in Table 2.

Table 2: Average and standard deviation of disagreement rate (r_d) among all 7 different algorithms for different training ratios.

Ratio	50%	70%	90%
r_d	29.16 ± 2.13%	24.31 ± 1.40%	20.12 ± 1.62%

As we can see from Table 2, the average rate of disagreement varies from 30% to 20% as the training ratio increases. Therefore, we have built a pool of 1050 (7 algorithms times 5 training ratios times 30 random partitions) diverse models, each of which achieves reasonably good classification performance.

4.1 With or Without Stage Range Information

In the current FlyExpress database, all the BDGP images are annotated with stage ranges. Clearly the additional stage range information can be used as prior knowledge to make the prediction of

exact stages more reliable. When no range information is available, the problem is a 15-class classification problem. With the additional stage range information, the problem can be reduced into a binary-class, 3-class or 5-class problem depending on which stage range the corresponding image belongs to.

We use similar experimental settings as in "Materials and Methods", and calculate the average classification performance when the stage range is given. The results are summarized in Table 3.

Table 3: Average classification accuracy on the validation set for different algorithms and different training ratios, when stage range information is available. 7 algorithms are evaluated, including SVM with linear kernel (SVM) and 6 sparse learning methods: Lasso (lasso), group Lasso (glasso), sparse group Lasso (sglasso) with least square loss (least) and logistic loss (log).

Ratio	50.00%	60.00%	70.00%	80.00%	90.00%
SVM	86.27%	86.31%	86.46%	86.87%	87.10%
lasso(least)	84.78%	85.18%	85.82%	86.34%	86.41%
glasso(least)	85.55%	85.85%	86.06%	86.49%	86.47%
sglasso(least)	85.53%	85.90%	86.01%	86.61%	86.88%
lasso(log)	85.34%	85.60%	85.72%	86.29%	86.57%
glasso(log)	85.44%	85.85%	85.99%	86.05%	86.46%
sglasso(log)	85.46%	85.72%	85.97%	86.32%	86.87%

As we can see from Table 3, when the stage range is given, the classification performance for all cases significantly improves. The only drawback of using the stage range information is that one cannot provide a complete prediction histogram for all 15 stages to perform sub-stage annotation (only the histogram for the corresponding stage range). In our study, we combine the results from both with and without range classifiers. For an un-annotated image, we first use with-range classifiers to determine the stage of this image, and then use the prediction histogram $\mathbf{h}$ of without-range classifiers to perform sub-stage annotation. For example, if the with-range classifiers decide that this image belongs to stage i, we will then use $\mathbf{h}[i-1]$, $\mathbf{h}[i]$ and $\mathbf{h}[i+1]$ to calculate the sub-stage and the decimal stage value.

In the previous evaluations, we use the overall accuracy across all stages to illustrate the performance of our system. It is also interesting to see the differences between stage ranges in terms of the annotation accuracy. We use the linear SVM classifier as an example to summarize the average annotation accuracies for all 5 different stage ranges with different training ratios. The results are presented in Table 4. We can conclude from Table 4 that the stage range with the best classification performance is stages 11-12, where the accuracy is as high as 97% when 90% of the data is used as training. The most challenging stage range for our system is stages 9-10, where only 76% of the time an accurate prediction is made. This is consistent with our previous experiments.

5 Ensemble Pruning

We have shown that combining different learning algorithms is beneficial. Next, we present another set of experiments where a subset of models (instead of a subset of algorithms) are selected to form the ensemble. This is often referred to as ensemble pruning. Two model selection methods are tested:

- **Random Subset**. In this setting, a number of models are randomly selected from the trained

Table 4: Average classification accuracy within each stage range for different training ratios. SVM with linear kernel is used as the classifier and stage range information is available.

Ratio	4-6	7-8	9-10	11-12	13-17
50.00%	86.43%	95.27%	74.10%	95.61%	83.64%
60.00%	86.08%	95.27%	75.13%	95.97%	83.48%
70.00%	87.05%	95.39%	74.08%	96.38%	83.53%
80.00%	87.33%	95.79%	75.23%	96.40%	83.89%
90.00%	87.70%	95.60%	76.47%	97.07%	82.76%

models to form the model ensemble. This process is repeated 1000 times to report the average result.

- **Ranked by Validation Accuracy**. In this setting, the trained 1050 models are first ranked by their classification accuracy on the validation set, with the most accurate one on the top of the list. Then, only the top few of the models are selected to form the model ensemble.

We vary the number of models used for the ensemble, and report the performance using the independent evaluation dataset in Table 5. As we can observe from Table 5, 30 models are sufficient to accurately annotate the stage of a given image. By increasing the number of models, the stage accuracy ($\text{Acc}_{\text{Stage}}$) and the plus-minus-half accuracy ($\text{Acc}_{\pm 0.5}$) remain stable while the sub-stage accuracy ($\text{Acc}_{0.5}$) steadily improves from 63% to 75.5%. Thus, for accurate sub-stage annotation, combining all models is essential.

Table 5: Performance evaluation of model ensemble using different subsets of the trained models. In the "Random Subset" category, a number of models are randomly selected from the trained models to form the model ensemble. In the "Ranked by Validation Accuracy" category, the trained models are first ranked by their accuracy on the corresponding validation set such that the top models can be selected. Only the case without stage range information is considered.

	Random Subset			Ranked by Validation Accuracy		
Number of Models	$\text{Acc}_{0.5}$	$\text{Acc}_{\text{Stage}}$	$\text{Acc}_{\pm 0.5}$	$\text{Acc}_{0.5}$	$\text{Acc}_{\text{Stage}}$	$\text{Acc}_{\pm 0.5}$
30	62.82%	81.63%	93.49%	59.83%	82.05%	92.31%
50	64.56%	81.65%	93.56%	62.39%	81.20%	93.16%
100	66.96%	81.58%	93.59%	62.39%	81.20%	94.02%
200	69.22%	81.49%	93.52%	62.39%	81.20%	94.87%
500	71.41%	81.42%	93.44%	66.67%	82.05%	94.87%
1050	73.50%	81.20%	93.16%	73.50%	81.20%	93.16%
